# Meta-network: optimized species-species network analysis for microbial communities

**DOI:** 10.1186/s12864-019-5471-1

**Published:** 2019-04-04

**Authors:** Pengshuo Yang, Shaojun Yu, Lin Cheng, Kang Ning

**Affiliations:** 1 Key Laboratory of Molecular Biophysics of the Ministry of Education, College of Life Science and Technology, Huazhong University of Science and Technology, Wuhan, 430074 Hubei China; 2 Department of Engineering, Trinity College, 300 Summit Street, Hartford, CT 06106 USA

**Keywords:** Microbial network, Data-mining, Network analysis, Associate-rule mining

## Abstract

**Background:**

The explosive growth of microbiome data provides ample opportunities to gain a better understanding of the microbes and their interactions in microbial communities. Given these massive data, optimized data mining methods become important and necessary to perform deep and comprehensive analysis. Among the various priorities for microbiome data mining, the examination of species-species co-occurrence patterns becomes one of the key themes in urgent need.

**Results:**

Hence, in this work, we propose the Meta-Network framework to lucubrate the microbial communities. Rooted in loose definitions of network (two species co-exist in a certain samples rather than all samples) as well as association rule mining (mining more complex forms of correlations like indirect correlation and mutual information), this framework outperforms other methods in restoring the microbial communities, based on two cohorts of microbial communities: (a) the loose definition strategy is capable to generate more reasonable relationships among species in the species-species co-occurrence network; (b) important species-species co-occurrence patterns could not be identified by other existing approaches, but could successfully generated by association rule mining.

**Conclusions:**

Results have shown that the species-species co-occurrence network we generated are much more informative than those based on traditional methods. Meta-Network has consistently constructed more meaningful networks with biologically important clusters, hubs, and provides a general approach towards deciphering the species-species co-occurrence networks.

**Electronic supplementary material:**

The online version of this article (10.1186/s12864-019-5471-1) contains supplementary material, which is available to authorized users.

## Background

Network-based approaches are gaining momentum as one of the most helpful tools for the analysis of microbial community structure. They offer new methodological and biological insights to investigate species interactions. Many microorganisms co-exist by interacting with each other and effectively exert various functions [1]. In addition, due to currently insufficient understanding of the community structure, the mounting volume of metagenomics data limits the traditional network analysis to recover the real relationships in bacterial community [2]. Hence finding out intricate yet important associations (for example, to explore the cyclical process of a substance or element in a bacterial community [3]) becomes increasingly challenging for traditional methods.

To address these limitations, we propose Meta-Network to establish the species-species co-occurrence network. The loose definition method is introduced first to recover more correlations before correlation calculation. Then we utilize the FS-Weight and PCA-PMI (Part Mutual Information adjusted by Path Consistency Algorithm) methods to explore the indirect and non-linear correlations, respectively. To investigate the optimized species-species co-occurrence network, systematic evaluation is investigated to discover meaningful biological implications in the species-species co-occurrence network.

## Method

### Construction workflow of species-species co-occurrence network

**Fig. 1:**
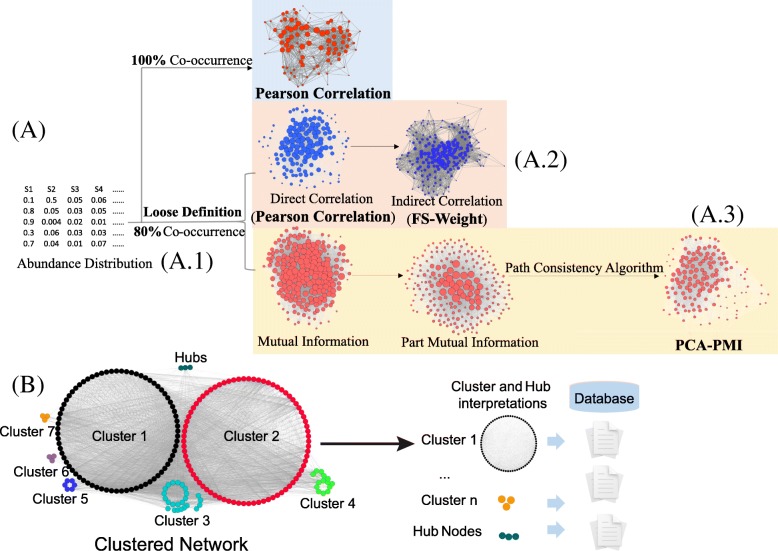
Network establishment and cluster analysis for species-species co-occurrence network. **a** Network construction. Based on the loose definition method (A.1), networks were constructed by two approaches. First, the direct correlations were detected and a graph-based algorithm (FS-Weight) was applied to detect indirect correlations (A.2). Second, the part mutual information was calculate, followed by a few rounds of adjustment based on Path Consistency Algorithm (PCA) until the network has no correlations added or removed (A.3). **b** Clustering analysis**.** Density MCODE clustering algorithm is applied to categorize the network into functionally or taxonomically enriched units. Hub nodes represent species connected to many other species and usually play key roles in the community. Databases curation and literature mining are used to annotate the clusters and hub nodes

While direct and linear correlations were calculated by Pearson and Spearman [4], limitations of these algorithms are developed in quantifying the relationship among species pairs: First, the correlations are calculated when pairwise species exist in all samples, which fail to recover the microbial communities owing to the sparse distribution of species. Second, Pearson and Spearman algorithms only capture direct and linear correlations for pairwise species, but cannot detect complex ecology correlations patterns [5]. In response to these observations, we applied several association rule mining algorithms to uncover complex correlations among species (for example, indirect correlation and non-linear correlations) (Fig. 1, a). First, we propose to recover correlations filtered by traditional methods, termed as the loose definition method (Fig. 1, a.2). Second, While direct relationships have been reported in many existing works in the context of intricate bacterial community, more complex correlations can be detected using association rule mining [6]. Hence we develop two methods to detect complex forms of correlations like indirect correlations (FS-Weight) and non-linear correlations (PCA-PMI) (Fig. 1, a.3, and a.4).

#### Loose definition of species-species relationship (Fig. 1, a.1)

**Fig. 2:**
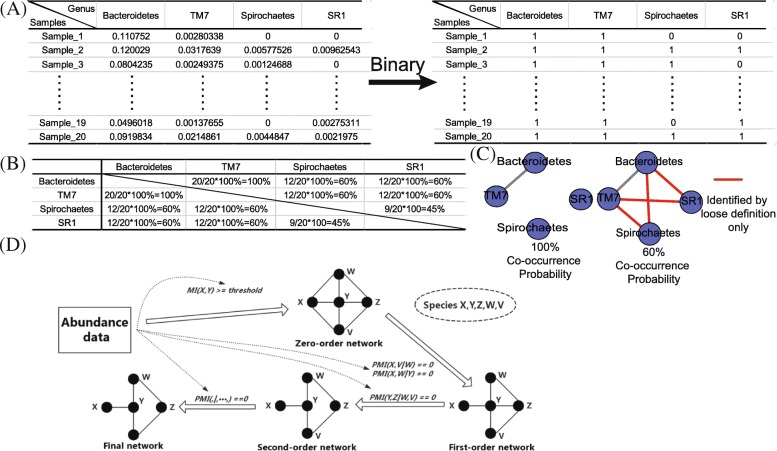
New methods for detecting potential correlations. **a** The presence–absence matrix is the binary form of original abundance matrix. In this matrix, columns present samples, rows present species, and entries are the presence (1) or absence (0) of a species. Samples are organized as a presence–absence matrix in which i = 1 to R rows and j = 1 to C columns. The entry a_ij_ in the matrix represents the presence (1) or absence (0) of species j in sample i. **b** Co-occurrence probability matrix for the sample data. In loose definition method, co-occurrence means two species in the same sample have both non-zero abundance. The co-occurrence probability of two species is defined as co-occurrence times / total sample count. **c** Network establishment based on different co-occurrence probability thresholds. Traditional network construction workflow only calculates correlations between species between 100% co-occurrence probability (left network), which is too strict for sparse distribution. More correlations are detected between co-occurrence probability set to 60% (right network, correlations detected by loose definition only were colored in red). **d** An overview of the PCA-PMI process. Species abundance distribution was calculated by the mutual information method and then the zero-order network, which contains all the mutual information, was constructed. PCA-PMI process was applied to adjust the zero-order network until no correlations changed

To measure the number of co-exist samples for pairwise species, we introduce the co-occurrence probability (Fig. 1, a.1). To be precise, we first convert the original abundance matrix into a presence-absence matrix, then we calculate the ratio of co-exist samples to all the samples as co-occurrence probability for each pairwise species (Fig. 2, a and b). When co-occurrence probability reaches above a user-defined threshold, the correlation for this pairwise species will be calculated (80% is used here as default, Fig. 2, c). It is worth mentioning that some genera pairs exist in a few number of samples but yield abnormally high correlation from Pearson and Spearman algorithms. However, no known literature and functionality can support this kind of correlation. Noise like this was filtered out based on the sample quantity set of loose definition.

#### Indirect relationships in the network (Fig. 1, a.2)

While direct relationships have been reported in many existing works in the context of intricate bacterial community, it is possible to detect more complex correlations using association rule mining [7]. In bacterial community, bacteria which do not interact but share interaction partners may play roles in the same functional pathway [8]. Hence, in our context, the FS-Weight method is applied to detect the indirect correlations (Fig. 1, a.2). FS-Weight measures the overlap between the pairwise species, and is originally designed to estimate association between direct and indirect correlations based on network structure [9].

For pairwise species, FS-Weight value between them was calculated in two steps. First, the direct correlations were calculated using a user-defined threshold for edge value. In this work, Pearson coefficient correlation was selected and the thresholds was set as 0.5 on genus level and 0.7 on OTU level based on previous research [10]. Second, the network constructed by FS-Weight was applied to filter out less reliable correlations and to add meaningful indirect relationships. FS-Weight threshold was set to 0.7 on OTU level and 0.5 on genus level based on the comparison between different thresholds. The benchmark result for FS-Weight on genus level was shown at Additional file 1: Figure S1, A and B.

#### Nonlinear associations (Fig. 1, a.3)

In microbial communities, nonlinear relationships play important roles [11]. Thus, to explore potentially nonlinear correlations, we adopt PCA-PMI method to explore non-linear correlations in microbial [12] (Fig. 2, d). PCA-PMI method calculate the partial information to measure the linear and non-linear relationship for each pairwise correlations in microbial community. PMI is defined as follows: assuming X and Y represent two one-dimensional variables representing abundance distribution in all the samples for species A and B respectively, and Z means an n-2 dimensional vector (n-2 > 0) representing other species abundance distribution in all the samples, the PMI between species x and y given indirect neighbor z is defined as below:

1 $$ \mathrm{PMI}\left(\mathrm{x},\kern0.5em \left.\mathrm{y}\right|\mathrm{z}\right)=\kern0.5em {\sum}_{\mathrm{x},\mathrm{y},\mathrm{z}}\mathrm{p}\left(\mathrm{x},\mathrm{y},\mathrm{z}\right)\log \kern0.5em \frac{p\left(\mathrm{x},\left.\mathrm{y}\right|\mathrm{z}\right)}{p^{\ast}\left(\left.\mathrm{x}\right|\mathrm{z}\right){p}^{\ast}\left(\left.\mathrm{y}\right|\mathrm{z}\right)} $$

Where the Part independence of species x and y given indirect neighbor z is defined as:

2 $$ {p}^{\ast}\left(\left.y\right|z\right)={\sum}_xp\left(\left.y\right|z,x\right)p(x) $$

3 $$ {p}^{\ast}\left(\left.x\right|z\right)={\sum}_yp\left(\left.x\right|z,y\right)p(y) $$

In order-one PCA-PMI, only one species was considered as indirect neighbor between two species, and the Path Consistency Algorithm (PCA) [13] was applied to adjust the correlation distribution using a user-defined correlation threshold (0.02 as default). This threshold was set based on the comparison between different thresholds. The benchmark result for FS-Weight on genus level was shown at Additional file 1: Figure S1, C and D. The sparse matrix is defined as a matrix owing a large number of nodes and sparse species distribution, thus it is feasible to use PCA method to construct the species-species co-occurrence network [14]. After all linear and non-linear correlations calculated, the network was updated by detecting higher orders of PMI (more species were set as indirect neighbor for two species). Higher orders of PMI are calculated to check the correlation reliability iteratively until no more edges are changed.

### Identification of clusters and hubs in the network

Detecting co-occurrence patterns and their biological significance (Fig. 1**,** b) supply an important material to understand the microbial network. First, we perform cluster calculation and hub nodes detection based on density clustering. We apply the MCODE cluster algorithm to detect the potential clusters [15]. Aligning cluster members against taxonomical or functional annotation databases can predict potential cluster functions and taxonomical compositions.

To identify hub nodes, we calculate the most connected nodes as candidates for hub nodes. These nodes are selected to perform Kruskal-Wallis test (test differences in network distribution before and after deleting hub node) to decide whether they are hub nodes. Finally, we interpret the clusters and hub nodes based on known taxonomical and functional databases and literatures.

### Systematic evaluation methods

In order to compare Pearson, loose definition, FS-Weight, PCA-PMI algorithm, the correlation coefficient calculated by loose definition which is implemented by the loose definition plus Pearson method.

First, we calculate the global network alignment by MAGNA++ [16] based on genetic algorithm. The alignment quality is measured by edge correctness (EC), induced conserved structure (ICS) and symmetric substructure score (S3) for each pair of networks. Then, after all nodes compared based on Jaccard index, we construct a similarity tree measured by the global similarity between any two networks been compared.

Secondly, these four algorithms were compared based on their topological structures including network similarity, global properties and local properties by compNet software [17]. Furthermore, we analyze the local properties for all taxon based on the R package igraph (http://igraph.org/r/), including coreness, degree, eigenvector centrality, eccentricity, betweeness and degree centrality.

Thirdly, we examined the correlation distribution using the same set of species as target. Based on top 100 abundant genera in gut microbiome datasets, four networks are constructed to investigate their edge distributions, which reflect the correlation difference. Then network motif distribution was investigated by mfinder (version 1.2) [18]. The motif size is set to 4, the query random network size set as 100, and other parameters are set as default. Network motif distribution was calculated in all sub networks and ranked by their frequency.

## Results and discussions

### Network construction based on human gut datasets

We applied the Meta-Network on a dataset of health young Chinese as our representation of human gut (MGP15838 in MG-RAST database) [19]. It consists of 314 healthy young adults, covering 20 rural and urban cohorts from 7 ethnic groups and 9 provinces throughout China. 5,102,015 high-quality sequences were generated and QIIME (version 1.91) [20] was applied to process this dataset. Finally, we obtained in total 24, 125 microbial OTUs. On genus level, we identified in total 2124 genera in which 102 genera process the relative abundance above 0.1%.

**Fig. 3:**
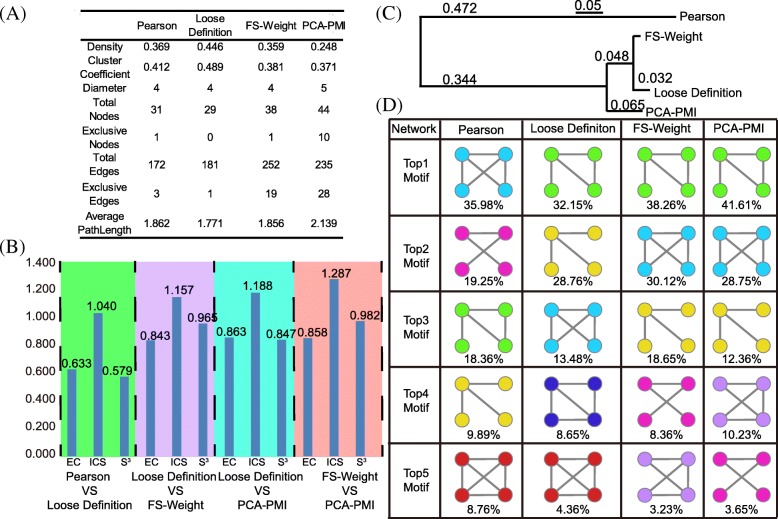
Network constructed and comparison among networks constructed by Pearson, Loose definition, FS-Weight and PCA-PMI methods on genus level. **a** Global properties for the four methods. The properties including network density, cluster coefficient, total nodes and edges, specific nodes and edges for each network and average path length were calculated. **b** Global network alignment result. Vertical axis represents the different alignment quality score. Horizontal axis represents the different pairwise alignments results. The quality score is ordered by edge correctness (EC), induced conserved structure (ICS) and symmetric substructure score (S^3^). **c** Network similarity tree constructed by Jaccard Index. FS-Weight algorithm was chosen as root. Based on different construction process, network constructed by FS-Weight and PCA-PMI has similar structures. **d** Motif distributions of four network construction methods. The four-node motif ranked with top 5 frequencies were illustrated. The percentage represented the proportion of motif in the entire 4-node-network motif, and each color indicated one specific motif in different network construction method

We compared networks constructed by all four algorithms (Pearson, Loose Definition, FS-Weight and PCA-PMI) on genus level. First, we calculated the global properties and illustrated in Fig. 3, a. Based on Pearson algorithm, 31 genera and 172 correlations were detected. Based on loose definition method, 29 genera and 287 correlations were detected. Network constructed by FS-Weight method detected 38 nodes and 252 edges. In network constructed by PCA-PMI method, 44 nodes and 235 edges were detected. Based on loose definition algorithm, the network removed 2 genera and 30 correlations, in which 81.16% correlations are among genera counted for average relative abundance less than 0.1%. On the other hand, 145 correlations are added among the genera which dominate in the microbial community (average abundance over 0.1%). This result indicates the importance roles of high-abundance species in microbial community, which is consistence with previous researches [21]. Furthermore, comparison between network constructed by loose definition method and FS-Weight and PCA-PMI methods shows that more complex forms of correlations have been detected by Meta-Network analysis. In our analysis workflow, both FS-Weight and PCA-PMI are calculated only based on Pearson Correlation coefficient. It would be interesting to see how it performs for network construction based on other pair-wise correlation analysis method under the Meta-Network workflow. Hence we applied the same workflow to construct the networks based on CCLasso [5], and the analysis results were provided in Additional file 1.

### Network comparison based on human gut datasets

We performed a systematic evaluation to compare the network constructed by Pearson, loose definition, FS-Weight and PCA-PMI algorithms. Global network alignment was carried to compare the network constructed by these four algorithms on OTU level (Fig. 3, a). The alignment between networks constructed by Pearson correlation (100% co-occurrence probability) and loose definition method (80% co-occurrence probability) measured the co-occurrence probability optimization. Owing to a low EC and S3 score (0.63, 0.579, respectively), the two networks had a low match. Network constructed by FS-Weight and PCA-PMI methods had the highest quality score (S3 score: 0.982), indicating that the network constructed by FS-Weight and PCA-PMI methods had the highest similarity. Network alignment results indicate that: First, both two association rule mining methods show a low match to network constructed by Pearson method. Second, two different methods show a high similarity.

Based on the similarity tree based on Jaccard index, network constructed by Pearson method had a low similarity with other three networks (Fig. 3, b). Network constructed by loose-definition and FS-Weight had the shortest distance because FS-Weight employed loose-definition abundance as input to find indirect correlations and PCA-PMI based network had a similar structural composition compared to network constructed by FS-Weight method.

Based on their motif distributions, we compared these four networks to reflect homology relationship (Fig. 3, c). Network constructed by loose definition and FS-Weight method present similar network motif distributions, and this result suggested a high homology [22]. Motifs detected in Pearson method had a different distribution with the others (Fig. 3, d).

### Correlation distribution and subnetwork comparison based on human gut datasets

**Fig. 4:**
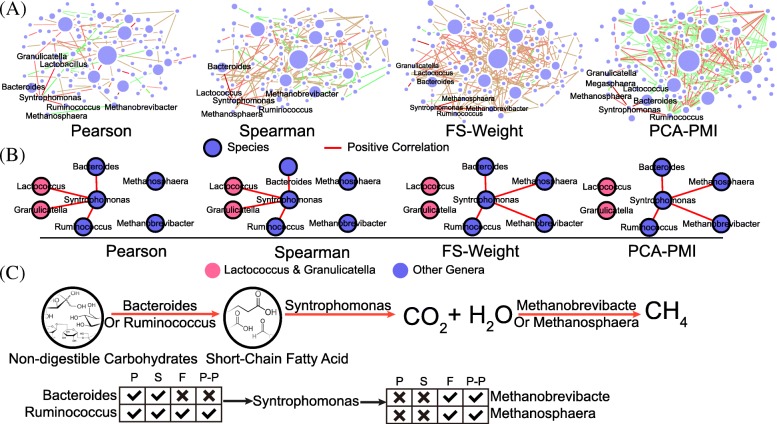
Subnetwork comparison for the network constructed by Pearson, Spearman, FS-Weight and PCA-PMI methods for human gut microbiome. **a** Network generated based on four methods based on top 100 gut genera. Genera whose average abundance ranked top 100 are selected, based on which the four methods were compared. **b** Correlation of genus in the same subnetwork. Pearson and Spearman method detect non-related correlations which could be filtered out by loose definition method. Based on indirect neighbor (FS-Weight) and non-linear correlations (PCA-PMI) calculations, previously hidden correlations (between *Syntrophomonas* and *Methanosphaera*, for example) could be discovered. In (**a**) and (**b**), each node represents a genus and the node size represented the average abundance in all samples. Edges indicates the correlations and red edges represent the positive correlations and green edges represent the negative correlations. **c** The possible interaction mechanism in the subnetwork for (**b**). Non-digestible carbohydrates could be degraded by *Bacteroides* and *Ruminococcus* to produce the short-chain fatty acid. Further reactions are generated between *Syntrophomonas* and methanogens to transfer the short-chain fatty acid into the methane. **d** Correlation distribution for four methods in non-digestible carbohydrate pathway. We calculated the correlations between *Syntrophomonas* and other genera (*Bacteroides, Ruminococcus, Methanosphaera, Methanobrevibacter*) detected by different methods (P: Pearson, S: Spearman, F: FS-Weight, P-P: PCA-PMI), and a tick represented the correlation was detected, and no detection was indicated by a cross

To investigate the edge distribution, four algorithms were compared based on the top 100 genera (Fig. 4, a). In network constructed by Pearson and Spearman algorithms, 52 and 63 correlations were identified among low abundance genera (illustrated as small node size). However, in network constructed by FS-Weight and PCA-PMI methods, most correlations were identified among the nodes counted as high abundance (over 0.1%). Furthermore, in view of the importance of abundance and function in microbial community, we can further speculate that these genera play important role in the gut microbiome [23].

In the network constructed by Pearson and Spearman algorithm (Fig. 4, b), Genera *Lactocuccus* and *Granulicatella* processed low abundance (average abundance 1.25e-5, 2.58e-4, respectively) and existed in 15 and 14 samples, respectively. In addition, a strong and significant positive correlation were detected between them (correlation coefficient 0.915, 0.823, respectively, both *p*-value < 0.01). No convincing literature and reported function (*Lactocuccus*: commonly identified as produce lactic acid, *Granulicatella*: commonly identified as potential pathogenic bacteria) could prove their relationships and both genera [19, 20]. These two correlations might be noise correlations and do not take part in this non-digestible carbohydrates degradation pathway.

More importantly, FS-Weight and PCA-PMI methods are capable to discover correlations undetected by the Pearson and Spearman algorithms (Fig. 4, d). For example, genus *Syntrophomonas* is reported as a fatty-acidoxidizing genus which cooperates with methanogens such as genus *Methanosphaera* and *Methanobrevibacter* [24]. However, Pearson method failed to detect these correlations, probably due to the large heterogeneity (different kinds of methanogens in gut samples) in human gut dataset. On the contrary, the correlations between *Syntrophomonas* and methanogens could be clearly identified by FS-Weight and PCA-PMI methods (FS-Weight correlation: 0.873, 0.926; PCA-PMI correlation: 0.912, 0.915). The interaction mechanism was illustrated in Fig. 4, c. First, genera such as *Bacteroides* and *Ruminococcus* degrade the non-digestible carbohydrates into the short-chain fatty acid [25]. Second, the *Syntrophomonas* and methanogens cooperate to transfer the short-chain fatty acid into methane and energy. Combining the network analysis results with literature review, we speculate that *Bacteroides* and *Ruminococcus* play the role, as indirect neighbor, between the *Syntrophomonas* and methanogens.

In network constructed by CCLasso, the indirect correlation pattern could also be found, which could again prove the indirect pattern and advantage of FS-Weight and PCA-PMI. The result is provided in Additional file 1.

### Network construction and analysis based on the Tara oceans datasets

**Fig. 5:**
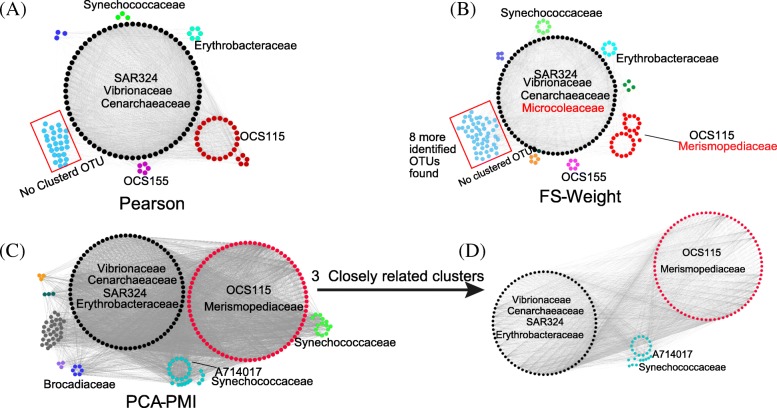
The modules generated based on networks constructed by different methods on OTU level for the *Tara* Oceans dataset. **a** Network constructed by Pearson methods with 100% co-occurrence probability. After performing loose definition of 80% co-occurrence probability and indirect relationships, more OTUs were detected in existing clusters and new clusters are detected for (**b**) Network constructed by FS-Weight method and (**c**) Network constructed by PCA-PMI method. **d** Three closely related clusters detected in the network based on PCA-PMI method. In all four panels, each cluster was composed by *phylogenetic*ally close species are labeled in the same color. Based on the calculation of identified OTUs on the family level, the most abundant members of every clusters are annotated

We applied the same workflow to *Tara* Oceans Project (PRJEB1787, also known as Project ERP001736 on EBI Metagenomics Portal). The processed nucleotide sequences from 245 experimental runs were publicly available, of which the volume exceeded 1.3 TB. Parallel-Meta (version 3.0) [26] was adopted to process this dataset to calculate the taxonomical abundance distribution on genus and OTU levels. Based on the Pearson algorithm, 154 nodes and 4289 edges were detected, and 6 clusters were identified by the MCODE cluster algorithm (Fig. 5, a). The largest cluster was mainly composed with the members of phylum *Proteobacteria*, which was confirmed as the largest phylum in the ocean bacterial community [27].

Figure 5, b illustrated the network constructed by FS-Weight algorithm, in which 190 nodes and 8135 edges were detected, including 3910 indirect correlations. Ten clusters were calculated and 6 of them were composed with similar taxonomical composition compared to the network constructed by Pearson method. New cluster members were identified as common members in the ocean bacterial community [28]. The network constructed by PCA-PMI method was composed with 237 nodes and 5861 edges, with a clustering coefficient of 0.666 (Fig. 5, c). *Nitrosopumilus* and *Candidatus_Scalindua* were detected only in network constructed by PCA-PMI method and were identified as common members in the ocean environment [29].

These results have again, proved that the Meta-Network workflow has the ability to discover more complex formats of correlations. Moreover, Networks constructed by two different methods (FS-Weight and PCA-PMI) shared many identical network members and clusters, indicating that both methods portrayed the complex formats of correlations in microbial communities.

## Conclusion

Species-species co-occurrence network is becoming one of the emerging fronts for microbiome research, largely due to the important ecological patterns it could reveal. However, previous methods are limited by only detecting the direct correlations among pairwise species. This work presents the Meta-Network method that optimized the construction, analysis and interpretation of the species-species co-occurrence network, focusing on three critical processes for species-species correlation analysis: we first employ loose definition to recover correlations missed by strict co-occurrence probability before calculating correlations. Based on these candidate correlations, we also develop two association rule mining methods for the recurrence of the real bacterial community network: a graph-based method FS-Weight to detect indirect correlations, PCA-PMI method to detect indirect correlations. Therefore, we believe that Meta-Network provides a general approach towards deciphering the species-species co-occurrence networks.

This method could be improved in several ways: in line with current gene expression network inferences, we can also improve its high-level associations (i.e., associations between two clusters of species). A complete species-species network, which provides the full picture of the regulation profile in the community, should also include viruses. All of these will be considered in our future work.

## Additional file

Additional file 1: **Figure S1.** Threshold selection for FS-Weight and PCA-PMI method. (A) Global network properties for networks constructed by FS-Weight method. (DOCX 354 kb)

## Abbreviations

OTU: Operational Taxonomic Unit
PCA: Path Consistency Algorithm
PMI: Part Mutual Information
