# Supplementary material for: Meta-network: optimized species-species network analysis for microbial communities
Source: BMC Genomics. 2019 Apr 4;20(Suppl 2):187. doi: 10.1186/s12864-019-5471-1 (PMC6457071; doi:10.1186/s12864-019-5471-1)
Supplement: Supplementary file 1 — Figure S1. Threshold selection for FS-Weight and PCA-PMI method. (A) Global network properties for networks constructed by FS-Weight method. (DOCX 354 kb) [file 12864_2019_5471_MOESM1_ESM.docx]

# Supplementary file for “Meta-Network: Optimized species-species network analysis for microbial communities”

Pengshuo Yang^1, $^, Shaojun Yu^1, $^, Lin Cheng^2^, Kang Ning^1, *^

^1^ Key Laboratory of Molecular Biophysics of the Ministry of Education, College of Life Science and Technology, Huazhong University of Science and Technology, Wuhan, Hubei 430074, China,

^2^ Department of Engineering, Trinity College, 300 Summit Street, Hartford, CT 06106, USA.

^$^ These authors contributed equally to this work.

^*^ To whom correspondence should be addressed. E-mail: [ningkang@hust.edu.cn](mailto:ningkang@hust.edu.cn)

# Network construction based on SparCC correlation based on human gut microbiome

In our analysis workflow, both FS-Weight and PCA-PMI algorithm were calculated only based on Pearson Correlation coefficient, thus construct networks based on other methods was necessary to complete our Meta-Network workflow. We performed CCLasso correlation [1], which infers the correlation relationship among members of microbial communities.

To compare network constructed by Pearson algorithm and CCLasso, same human gut microbiome dataset was selected: health young Chinese as our representation of human gut (MGP15838 in MG-RAST database) [2]. On genus level, 2,124 genera were identified in which 102 genera process the relative abundance above 0.1%.

Based on CCLasso correlation, 42 genera and 201 correlations (31 genera and 172 correlations were detected in network constructed by Pearson correlation) were detected.

Based on loose definition method, 38 genera and 312 correlations were detected based on CCLasso. Compared to the network constructed by Pearson correlations, 29 genera and 287 correlations were detected. After detecting indirect correlations by FS-Weight, network constructed by CCLasso detected 43 nodes and 289 edges. In network constructed by Pearson correlaitons,38 nodes and 252 edges were detected. These results indicated that network constructed by CCLasso is more informative and complex.

We also checked the correlation among *Bacteroides*, *Ruminococcus*, *Syntrophomonas* and methanogens. In network constructed in CCLasso, week correlation is detected between *Bacteroides*, *Ruminococcus* (0.421). However, correlations between *Syntrophomonas* and methanogens were undetectable. By FS-Weight method, the correlations between *Syntrophomonas* and methanogens could be identified by FS-Weight methods (FS-Weight correlation: 0.915, 0.936).

The result is the same as networks constructed based on Pearson network. Hence, we speculate that *Bacteroides* and *Ruminococcus* play the role, as indirect neighbor, between the *Syntrophomonas* and methanogens.

Furthermore, these result again proved that more complex forms of correlations have been detected by Meta-Network analysis.

# Threshold Selection for FS-Weight and PCA-PMI

For applying the FS-Weight and PCA-PMI methods in the construction of microbial species-species co-occurrence network, none of previous research could select the best thresholds. Hence, to select the feasible threshold for microbial species-species co-occurrence network, we compare the network constructed by different threshold with the dataset of healthy gut microbiota of young Chinese population mentioned in our manuscript (MGP15838 in MG-RAST database) [2]. Benchmark result on genus level was shown in **Figure 1**.

FS-Weight was calculated in two steps. First, with previous research, Pearson coefficient correlation was selected to calculate direct correlation and the thresholds was set as 0.5 on genus level and 0.7 on OTU level[3, 4]. Second, the network modified by FS-Weight was applied to filter out less reliable correlations and to add meaningful indirect relationships. The network was constructed based on the threshold ranged from 0.1-0.9 and result was shown at **Figure 1, A.** With the threshold become bigger, the number of node and edge decrease. There is a sudden drop at 0.5 for global properties like cluster coefficient, network density. Moreover, with MCODE cluster algorithm [5], the network constructed based on the threshold 0.5 detected 5 clusters (**Figure 1, B**). Overall, we speculate that 0.5 is a good threshold for FS-weight to construct species-species co-occurrence network.

For PCA-PMI, the network was constructed based on the threshold ranged from 0.001-0.1 shown at **Figure 1, C**. With the threshold become bigger, the number of node and edge decrease. There is a sudden drop at 0.02 for global properties like cluster coefficient, network density. Moreover, with MCODE cluster algorithm [5], the network constructed based on the threshold ranged from 0.02-0.06 detected 5 clusters (**Figure 1, D**). Hence, it is feasible to choose the network with the biggest node and edge distribution for the threshold 0.02.


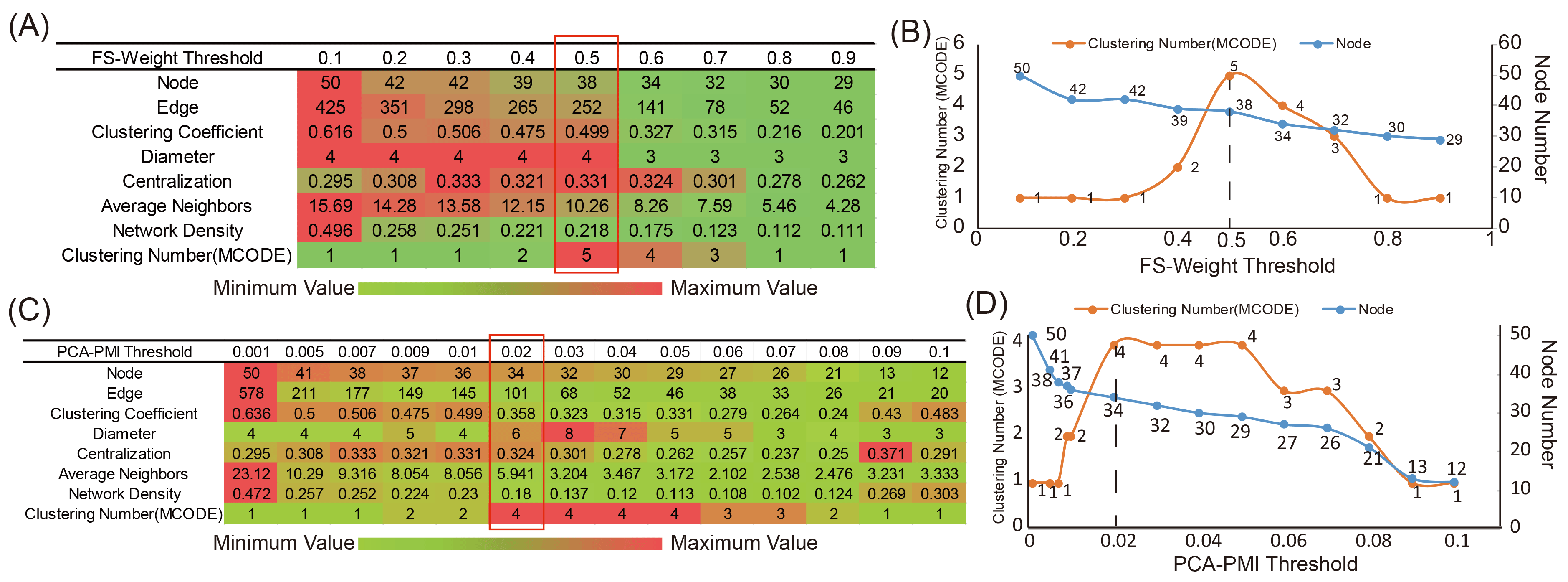


**Figure 1, threshold selection for FS-Weight and PCA-PMI method. (A)Global network properties for networks constructed by FS-Weight method.** Each column represents the network constructed by corresponding threshold. Each row means a global property. And the color of grid means the relative magnitude for certain global property. Threshold framed in red is chosen in our analysis. **(B)The node and cluster number distribution for network constructed by FS-Weight.** Curve labeled in blue is the node number distribution for network constructed by FS-Weight method. Curve labeled in orange is the clustering number distribution for network constructed by FS-Weight method. **(C)Global network properties for networks constructed by PCA-PMI method.** Each column represents the network constructed by corresponding threshold. Each row means a global property. And the color of grid means the relative magnitude for certain global property. Threshold framed in red is chosen in our analysis. **(D)The node and cluster number distribution for network constructed by PCA-PMI method.** Curve labeled in blue is the node number distribution for network constructed by FS-Weight method. Curve labeled in orange is the clustering number distribution for network constructed by FS-Weight method.

# Reference

1. Fang, H., et al., *CCLasso: correlation inference for compositional data through Lasso.* Bioinformatics, 2015. **31**(19): p. 3172-80.

2. Zhang, J., et al., *A phylo-functional core of gut microbiota in healthy young Chinese cohorts across lifestyles, geography and ethnicities.* ISME J, 2015. **9**(9): p. 1979-90.

3. Barberan, A., et al., *Using network analysis to explore co-occurrence patterns in soil microbial communities.* ISME J, 2012. **6**(2): p. 343-51.

4. Chaffron, S., et al., *A global network of coexisting microbes from environmental and whole-genome sequence data.* Genome Res, 2010. **20**(7): p. 947-59.

5. Wang, J., et al., *ClusterViz: A Cytoscape APP for Cluster Analysis of Biological Network.* IEEE/ACM Trans Comput Biol Bioinform, 2015. **12**(4): p. 815-22.
